# Supplementary material for: Munc18-1 is crucial to overcome the inhibition of synaptic vesicle fusion by αSNAP
Source: Nat Commun. 2019 Sep 23;10:4326. doi: 10.1038/s41467-019-12188-4 (PMC6757032; doi:10.1038/s41467-019-12188-4)
Supplement: Supplementary file 1 — Supplementary Information [file 41467_2019_12188_MOESM1_ESM.pdf]

SUPPLEMENTARY INFORMATION

Munc18-1 is crucial to overcome the inhibition of synaptic vesicle fusion by αSNAP

Stepien et al.

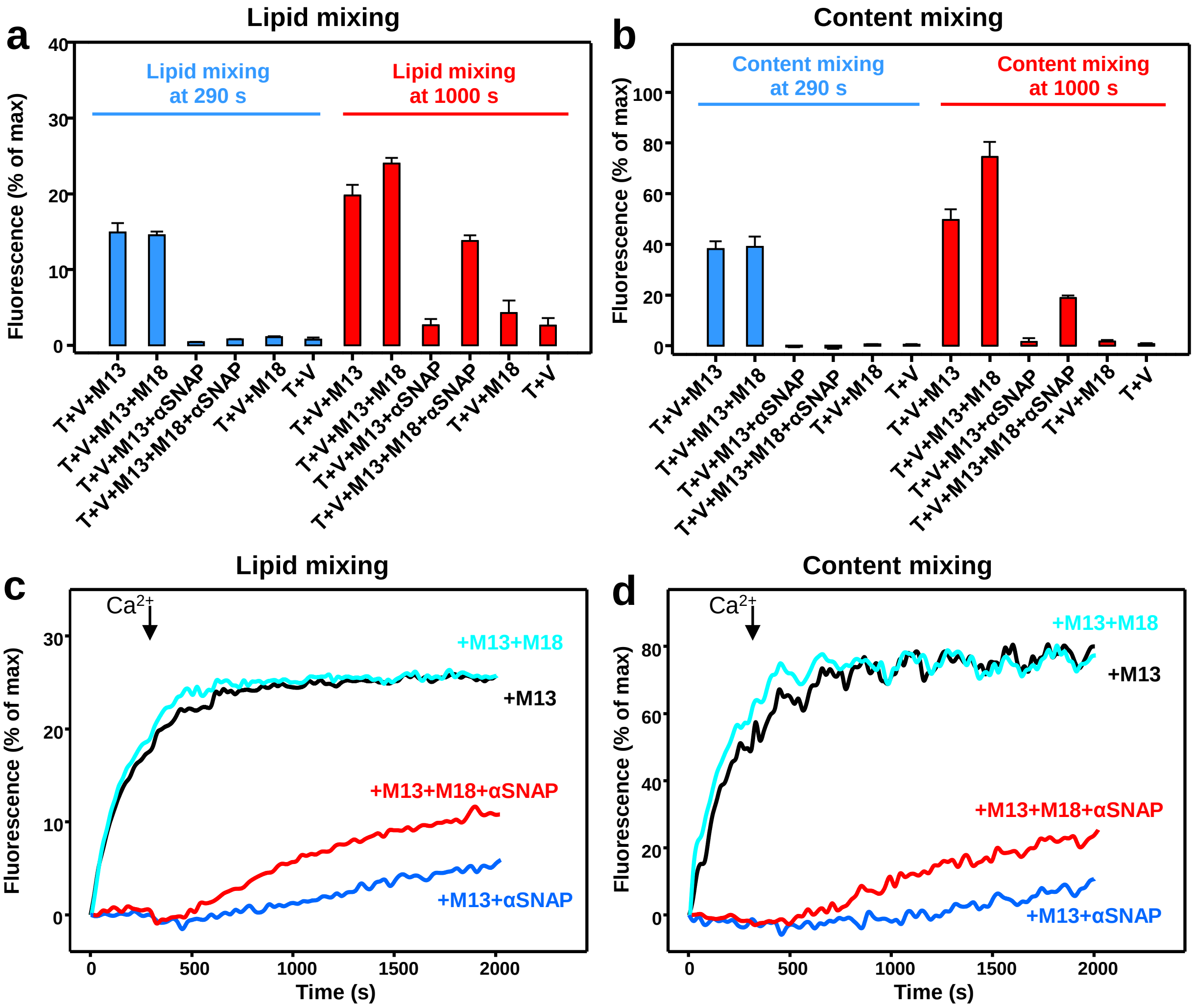

Supplementary Figure 1. Quantification of the fusion assays shown in Fig. 2a,b. **a,b** Bars represent averages of the normalized fluorescence intensities observed in lipid mixing (**a**) and content mixing (**b**) assays at 290 s (i.e. before Ca^{2+} addition) and at 1,000 s, performed in triplicates. Error bars represent standard deviations. Statistical analyses of the data are presented in Supplementary Data 1. **c,d** Lipid and content mixing assays performed under conditions analogous to those of Fig. 2a,b but using different preparations of V- and T-liposomes to illustrate the variability of the relative amplitude of the Ca^{2+}-dependent component of liposome fusion. Source data are provided as a Source Data file.

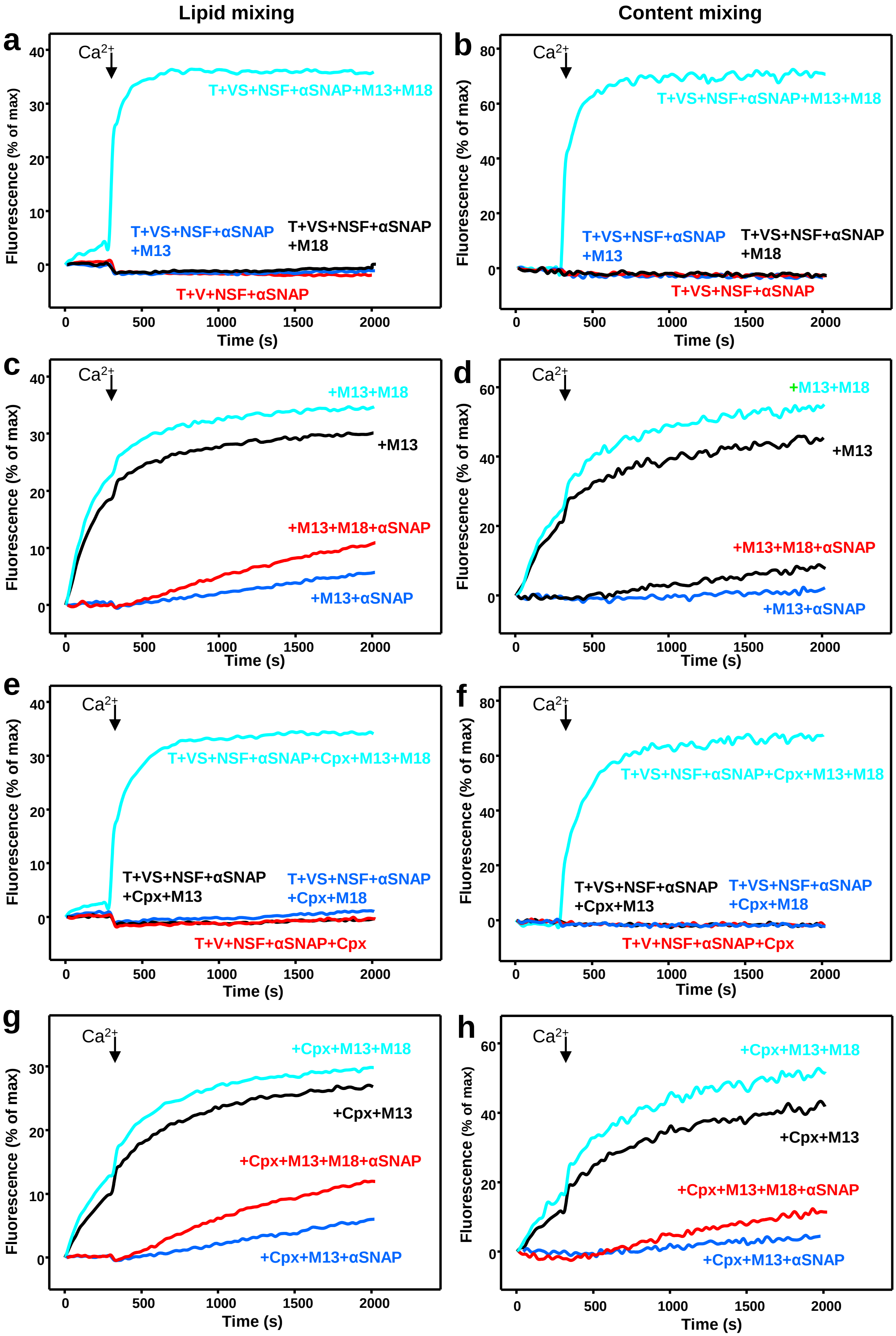
Lipid mixing
Content mixing
a
Ca2+
T+VS+NSF+αSNAP+M13+M18
T+VS+NSF+αSNAP
+M13
T+VS+NSF+αSNAP
+M18
T+V+NSF+αSNAP
Fluorescence (% of max)
Time (s)
b
Ca2+
T+VS+NSF+αSNAP+M13+M18
T+VS+NSF+αSNAP
+M13
T+VS+NSF+αSNAP
+M18
T+VS+NSF+αSNAP
Fluorescence (% of max)
Time (s)
c
Ca2+
+M13+M18
+M13
+M13+M18+αSNAP
+M13+αSNAP
Fluorescence (% of max)
Time (s)
d
Ca2+
+M13+M18
+M13
+M13+M18+αSNAP
+M13+αSNAP
Fluorescence (% of max)
Time (s)
e
Ca2+
T+VS+NSF+αSNAP+Cpx+M13+M18
T+VS+NSF+αSNAP
+Cpx+M13
T+VS+NSF+αSNAP
+Cpx+M18
T+V+NSF+αSNAP+Cpx
Fluorescence (% of max)
Time (s)
f
Ca2+
T+VS+NSF+αSNAP+Cpx+M13+M18
T+VS+NSF+αSNAP
+Cpx+M13
T+VS+NSF+αSNAP
+Cpx+M18
T+V+NSF+αSNAP+Cpx
Fluorescence (% of max)
Time (s)
g
Ca2+
+Cpx+M13+M18
+Cpx+M13
+Cpx+M13+M18+αSNAP
+Cpx+M13+αSNAP
Fluorescence (% of max)
Time (s)
h
Ca2+
+Cpx+M13+M18
+Cpx+M13
+Cpx+M13+M18+αSNAP
+Cpx+M13+αSNAP
Fluorescence (% of max)
Time (s)

Supplementary Figure 2. Synaptotagmin-1 and complexin-1 do not alter the inhibition of fusion between T- and V-liposomes caused by αSNAP. **a-h** Lipid mixing (**a,c,e,g**) of liposomes containing synaptobrevin and synaptotagmin-1 (VS-liposomes) with T-liposomes was monitored from the fluorescence de-quenching of Marina Blue lipids and content mixing (**b,d,f,h**) was monitored from the increase in the fluorescence signal of Cy5-streptavidin trapped in the VS-liposomes caused by FRET with PhycoE-biotin trapped in the T-liposomes upon liposome fusion. In **a-b**, assays were performed in the presence of NSF and αSNAP without (T+VS) or with Munc18-1 (M18) and/or M13C_1C_2BMUNC_2C (M13) as indicated by the color-coded labels. Assays in **c,d**, were performed under analogous conditions, but excluding NSF and including different combinations of Munc18-1, M13C_1C_2BMUNC_2C and αSNAP. Assays in **e-h** were performed under analogous conditions as those of **a-d**, but including 2 μM complexin-1 (Cpx). Experiments were started in the presence of 100 μM EGTA, 1 μM excess of SNAP-25 and 5 μM streptavidin, and Ca^{2+} (600 μM) was added at 300 s. Source data are provided as a Source Data file.

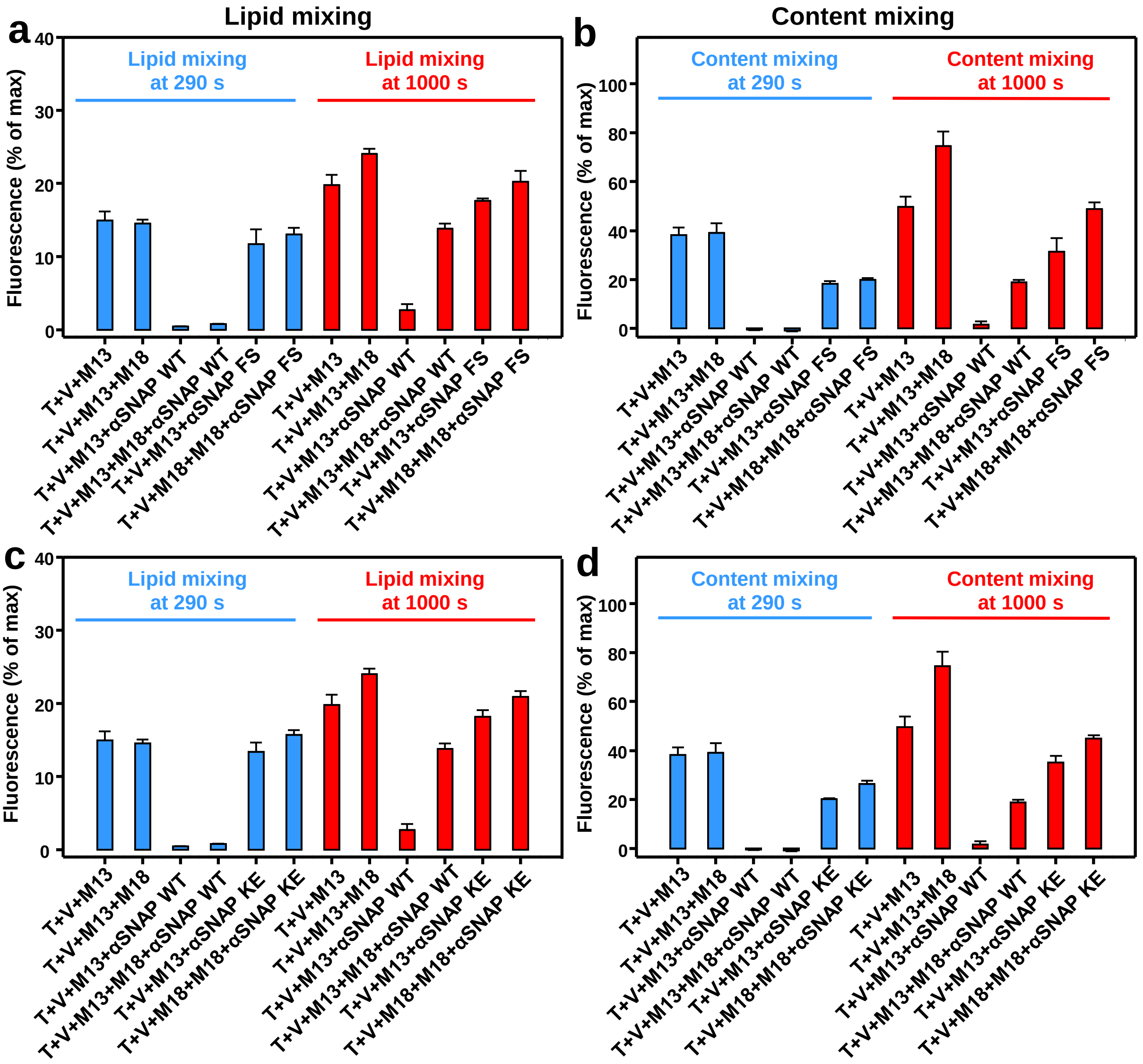

Supplementary Figure 3. Quantification of the fusion assays shown in Fig. 3. Panels **a-d** correspond to the experiments shown in panels **a-d** of Fig. 3, respectively. Bars represent averages of the normalized fluorescence intensities observed in lipid mixing (**a,c**) and content mixing (**b,d**) assays at 290 s (i.e. before Ca^{2+} addition) and at 1,000 s, performed in triplicates. Error bars represent standard deviations. Statistical analyses of the data are presented in Supplementary Data 1.

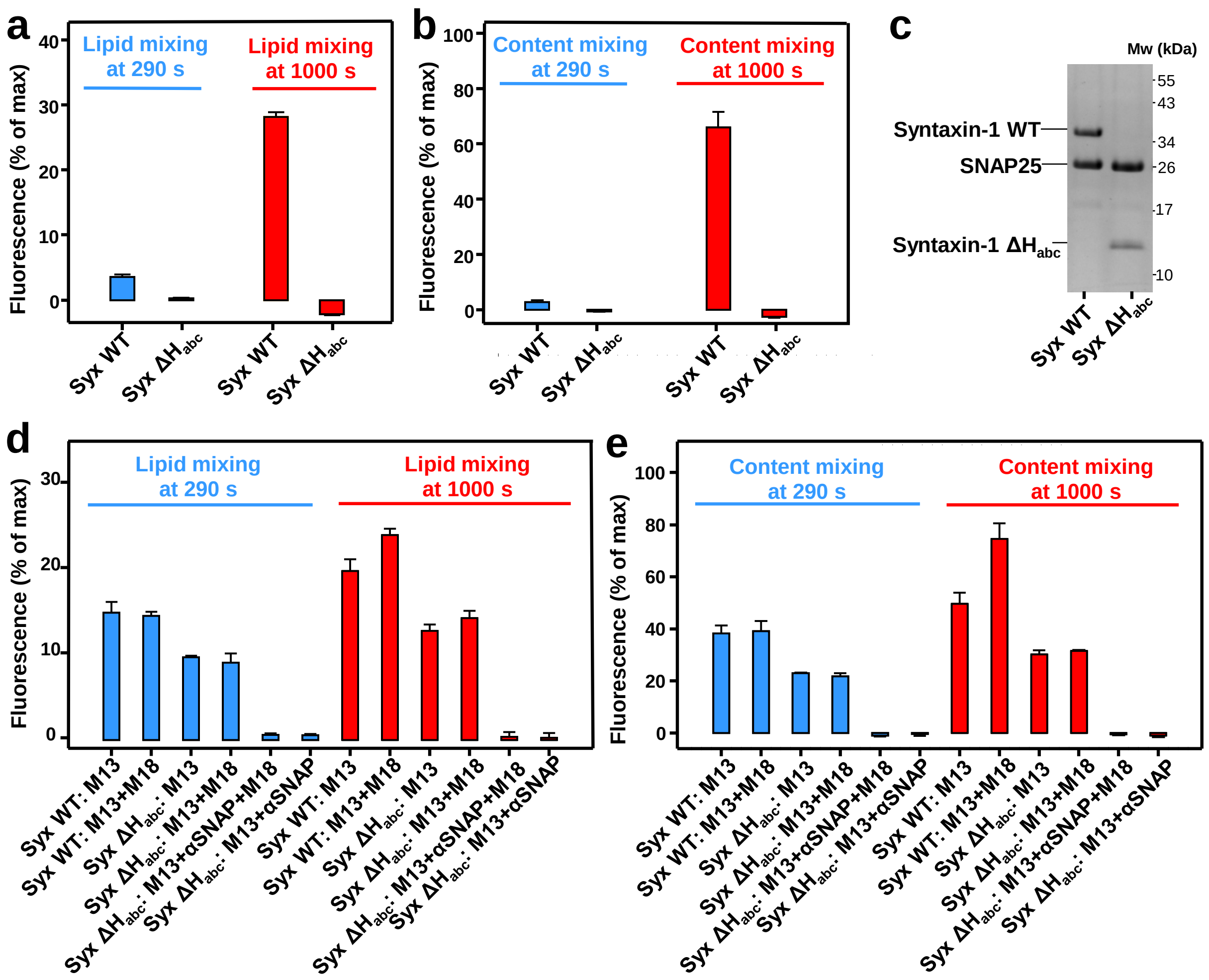

Supplementary Figure 4. Quantification of the fusion assays shown in Fig. 5. Panels **a,b,d,e** correspond to the experiments shown in panels **b-e** of Fig. 5, respectively. Bars represent averages of the normalized fluorescence intensities observed in lipid mixing (**a,d**) and content mixing (**b,e**) assays at 290 s (i.e. before Ca^{2+} addition) and at 1,000 s, performed in triplicates. Error bars represent standard deviations. Statistical analyses of the data are presented in Supplementary Data 1. **c** SDS PAGE analysis of T-liposomes containing SNAP-25 and WT or ΔH_{abc} syntaxin-1. Bands were visualized by Coomassie blue staining.

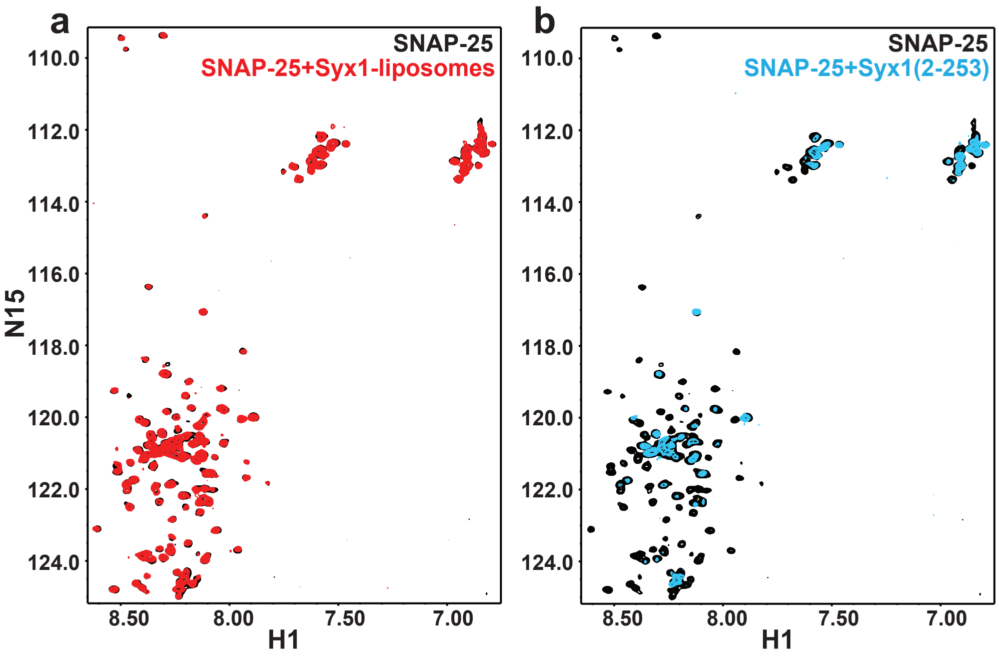

Supplementary Figure 5. SNAP-25 does not bind to liposome-anchored syntaxin-1. The contour plots show superpositions of ^{1}H-^{15}N HSQC spectra of ^{15}N-labeled SNAP-25 alone (black contours) or in the presence of S-liposomes (**a**, red contours) or syntaxin-1(2-253) (**b**, blue contours). The strong broadening of many cross-peaks caused by syntaxin-1(2-253) reflects its binding to SNAP-25, whereas no such broadening was caused by the S-liposomes, showing that liposome-anchored syntaxin-1 does not bind to SNAP-25.

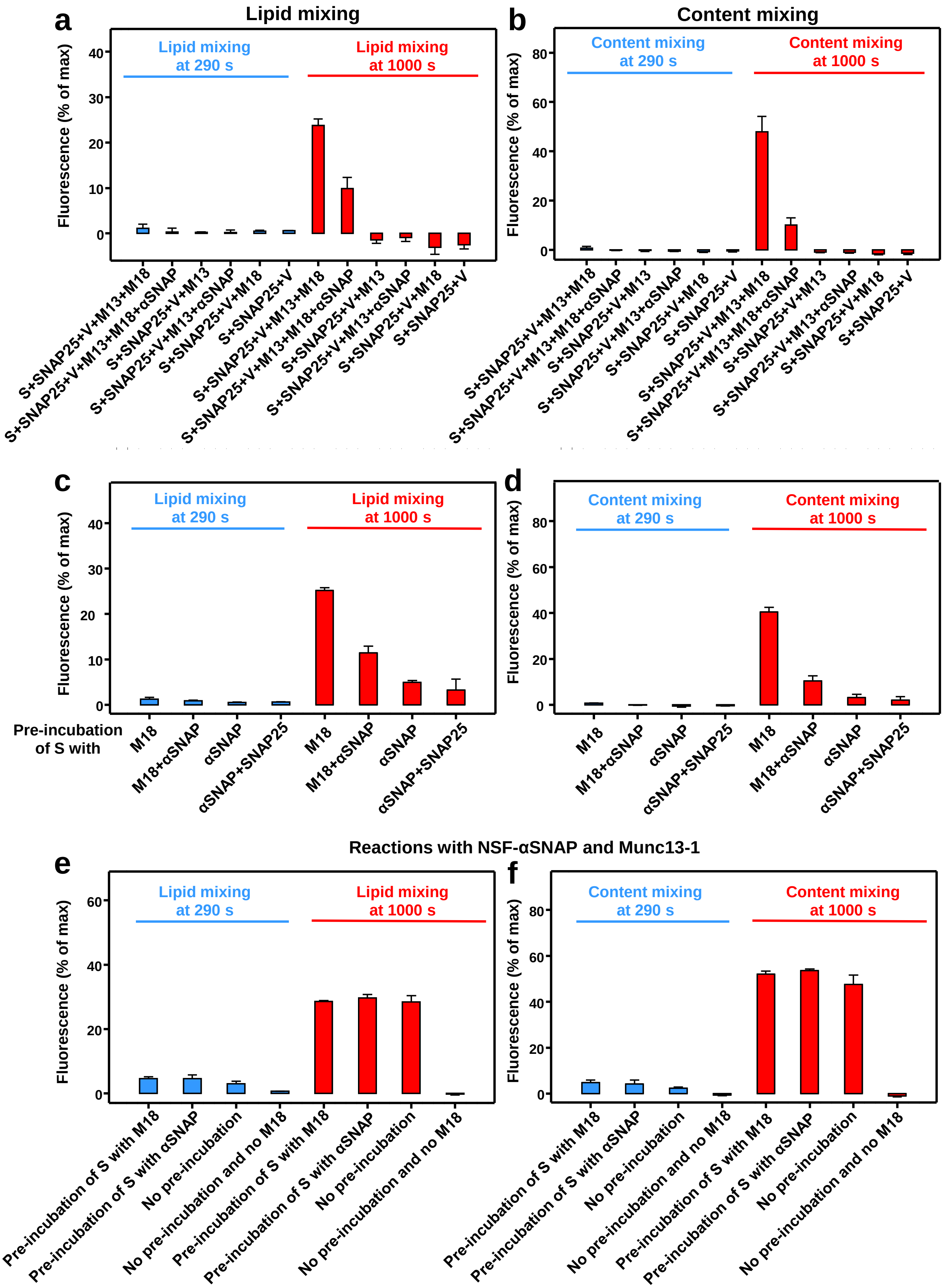
a
Lipid mixing
Lipid mixing at 290 s
Lipid mixing at 1000 s
Fluorescence (% of max)
S+SNAP25+V+M13+M18
S+SNAP25+V+M13+M18+αSNAP
S+SNAP25+V+M13
S+SNAP25+V+M13+αSNAP
S+SNAP25+V+M18
S+SNAP25+V
b
Content mixing
Content mixing at 290 s
Content mixing at 1000 s
c
Pre-incubation of S with
M18
M18+αSNAP
αSNAP
αSNAP+SNAP25
d
Reactions with NSF-αSNAP and Munc13-1
e
f
Pre-incubation of S with M18
Pre-incubation of S with αSNAP
No pre-incubation
No pre-incubation and no M18

Supplementary Figure 6. Quantification of the fusion assays shown in Fig. 7. Panels **a-f** correspond to the experiments shown in panels **a-f** of Fig. 7, respectively. Bars represent averages of the normalized fluorescence intensities observed in lipid mixing (**a,c,e**) and content mixing (**b,d,f**) assays at 290 s (i.e. before Ca^{2+} addition) and at 1,000 s, performed in triplicates. Error bars represent standard deviations. Statistical analyses of the data are presented in Supplementary Data 1.

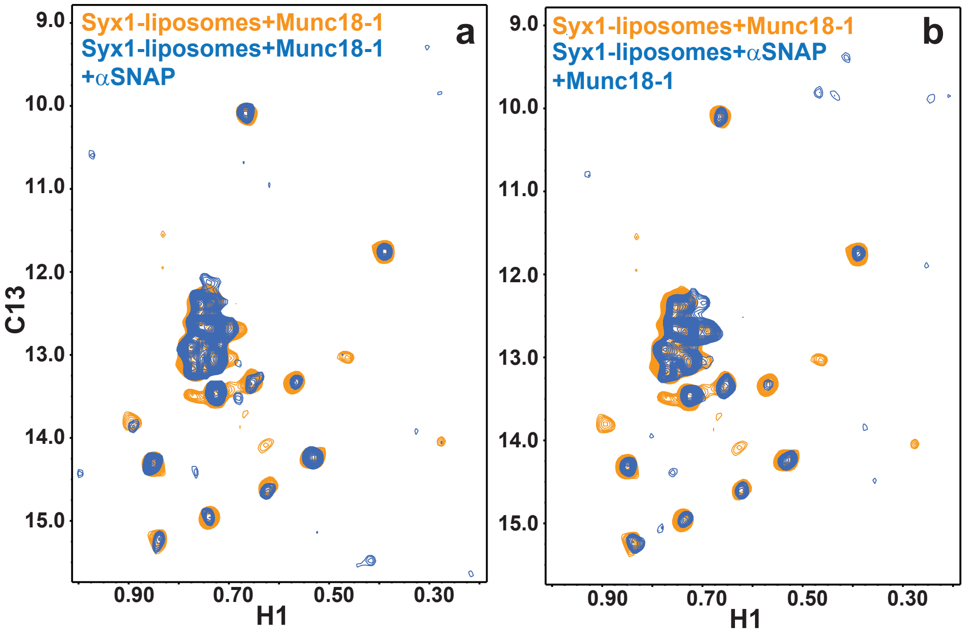

Supplementary Figure 7. Competition between Munc18-1 and αSNAP for binding to liposome-anchored syntaxin-1 monitored with ^{1}H-^{13}C HMQC spectra. The contour plots show superpositions of ^{1}H-^{13}C HMQC spectra of S-liposome containing ^{2}H-I-$^{13}CH_3$-syntaxin-1 bound to Munc18-1 (orange contours) and of S-liposome containing ^{2}H-I-$^{13}CH_3$-syntaxin-1 that were first incubated with Munc18-1 and then αSNAP (**a**, blue contours) or first with αSNAP and then with Munc18-1 (**b**, blue contours).

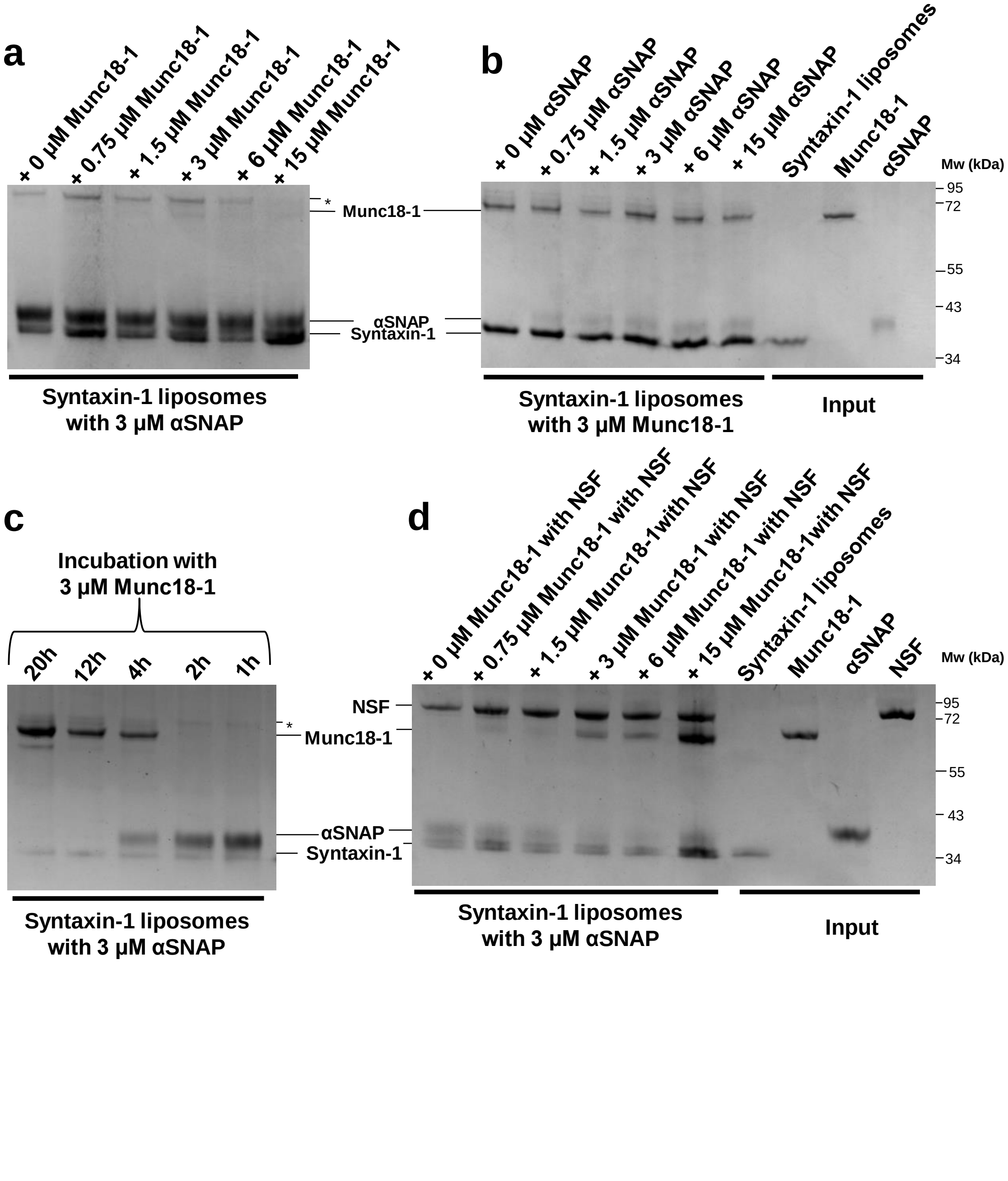

Supplementary Figure 8
Stepien et al.

Supplementary Figure 8. Munc18-1 and αSNAP compete for binding to liposome-anchored syntaxin-1. **a-d** Liposome co-floatation assays performed under the same conditions of the experiments shown in Fig. 9a-d, respectively. The positions of the various proteins and molecular weight markers are indicated. The * indicates a band that we observed occasionally and likely corresponds to syntaxin-1 dimerized in the gel. The gels illustrate the variability in some of the results of the co-floatation assays and at the same time the consistency of the overall data. Variability was observed for instance in the intensity of the bands observed in lanes expected to contain the same amount of protein (e.g. the syntaxin-1 bands in the different lanes of panel **a**), in the relative amounts of the bands from different proteins (e.g. compare the relative intensities of the syntaxin-1 and αSNAP bands in panel **c** with those of Fig. 9c), or in the amounts of Munc18-1 binding observed in experiments that included NSF (e.g. compare lanes 2 and 3 of panel **d** with those of Fig. 9d). These different types of variability can be attributed in part to technical difficulties associated with pipetting the entire layer containing the liposomes after co-floatation without mixing it with other layers, and to the non-linearity of the band intensity resulting from Coomassie staining. We also observed variability in the level of Coomassie blue staining of the proteins in the gels, which appears to be affected by the presence of lipids in the samples, particularly for syntaxin-1. Some variability may have also arisen because of differences in the amount of syntaxin-1 incorporated into the liposomes in different preparations and the strong tendency of membrane-anchored syntaxin-1 to aggregate[1]. Note however that, despite the variability, the same trends were observed in the experiments of panels **a-d** and Fig. 9, as well as multiple additional experiments performed under analogous conditions. Hence, these assays support the conclusions drawn about the competition between αSNAP and Munc18-1 for binding to syntaxin-1, as well as the predominant binding of Munc18-1 over αSNAP when samples are incubated for a sufficiently long time or contain NSF.

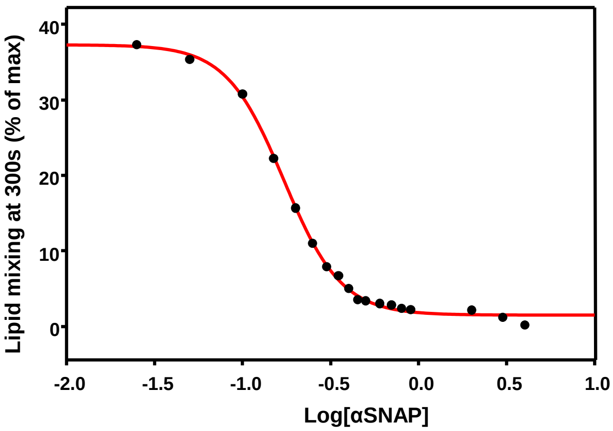

Supplementary Figure 9. aSNAP inhibits lipid mixing mediated by pre-formed trans-SNARE complexes with high affinity and cooperativity. The fluorescence intensity observed at 300 s in the lipid mixing assays shown in Fig. 10e was plotted as a function of the logarithm of the αSNAP concentration expressed in mM units. Fitting to a sigmoidal dose-response equation with Sigma Plot (Systat Software, Inc.) yielded an EC50 of 170 nM and a Hill coefficient of 2.66. Source data are provided as a Source Data file.

Supplementary Table 1. Primers used to generate the mutant proteins used in this study.

PCR oligo name	Description	Sequence	
		**Foward primer**	**Reverse primer**
Habc	Deletion of H_{abc} domain from full-length syntaxin-1A	GGACAGCGATGACGACGATGA TGTCCAGCCCTCAGTGAGATCG AGACC	GGTCTCGATCTCACTGAGGGC CTGGACATCATCGTCGTCATC GCTGTCC
C145A	Mutation C145A in full-length syntaxin-1A	GACTACCGAGAACGCGCGAAA GGGCGCTCCAG	CTGGATGCGCCCTTTCGCGCG TTCTCGGTAGC
C271A_C272A	Mutations C271A and C272A in full-length syntaxin-1A	GATCATGATCATCATTGCGGCG GTGATTCTGGGCATC	GATGCCCAGAATCACCGCCGC AATGATGATCATGATC
S186C	Mutation S186C in cysteine-free syntaxin-1A	ATCATCATGGACTCCTGCATCTC GAAGCAGGCC	GGCCTGCTTCGAGATGCAGGA GTCCATGATGAT
C103A	Mutation C103A in full-length synaptobrevin-2	CATCTTGGGAGTGATTGCGGCC ATCATCCTCATC	GATGAGGATGATGGCCGCAA TCACTCCCAAGATG
L26C	Mutation L26C in cysteine-free full-length synaptobrevin-2	GCACCTCCTCCAAATTGCACCA GTAACAGGAGACTG	CAGTCTCCTGTTACTGGTGCA ATTTGGAGGAGGTGC
M71D	Mutation M71D in cysteine-free full-length SNAP-25a	CATATCAACCAAGACGATAAGG AGGCCGG	CTCGGCCTCCTTATCGTCTTGG TTGATAG
L78D	Mutation L278D in cysteine-free full-length SNAP-25a	GAGGCCGAGAAAAATGATAAA GATTTAGGCAAATCC	GGATTTGCCTAAATCTTTATCA TTTTTCTCGGCCTC
K122E	Mutation K122E in full-length αSNAP	CGCTTCACCATCGCGGCCGAGC ACCACATC TCCATCGCC	GGCGATGGAGATGTGGTGCT CGGCCGCGATGGTGAAGCG
K163E	Mutation K163C in full-length αSNAP	GCAGTCCGCAGACTACTACGAA GGCGAGGAGTCCAACAGC	GCTGTTGGACTCCTCGCCTTC GTAGTA GTCTGCGGACTGC
F27S_F28S	Mutations F27S and F28S in full-length αSNAP	GAAGAACTCGCAGTCCTCCTCC TCGGGCCTCTTCGGAGG	CCTCCGAAGAGGCCCGAGGA GGAG GACTGCGAGTTCTTC
CHisLin	Insertion of LRLPETGSGSHHHHHHAA at the C-terminus of full-length αSNAP	GGAAGACCTGCGCCTGCCAGA AACCGGTTCAGGCTCACATCAT CACCATCACCACGCCGCCTAAG CTTAATTCATCG	CGATGAATTAAGCTTAGGCGG CGTGGTGATGGTGATGATGT GAGCCTGAACCGGTTTCTGGC AGGCGCAGGTCTTCC

Supplementary References

1. Weninger, K., Bowen, M.E., Choi, U.B., Chu, S. & Brunger, A.T. Accessory Proteins Stabilize the Acceptor Complex for Synaptobrevin, the 1:1 Syntaxin/SNAP-25 Complex. *Structure* **16**, 308-320 (2008).
