# Supplementary material for: Munc18-1 is crucial to overcome the inhibition of synaptic vesicle fusion by αSNAP
Source: Nat Commun. 2019 Sep 23;10:4326. doi: 10.1038/s41467-019-12188-4 (PMC6757032; doi:10.1038/s41467-019-12188-4)
Supplement: Supplementary file 3 — Description of Additional Supplementary Files [file 41467_2019_12188_MOESM3_ESM.pdf]

Description of Additional Supplementary Files

File name: Supplementary Data 1

Description: Statistical analysis of lipid and content mixing data. One-way ANOVA (Holm-Sidak method, as implemented in Sigma Plot) was used to analyze the statistical significance of the quantitative results of lipid and content mixing at 290 s and 1,000 s shown in Supplementary Figs. 1a,b, 3a-d, 4a,b,d,e, and 6a-f. For each group of data, the table describes the N values, the missing values, the means, the standard deviations (Std Dev), the standard error of the mean (SEM), the degrees of freedom (DF), the sum of squares (SS), the mean of Squares (MS), the F test statistic (F), the p value for the group (P), and, for each pairwise comparison, the difference of the means, the t values (t), the unadjusted p values (P), the critical level and the statistical significance.
